# Lymphatic Microsurgical Preventive Healing Approach (LYMPHA) for Lymphedema Prevention after Axillary Lymph Node Dissection—A Single Institution Experience and Feasibility of Technique

**DOI:** 10.3390/jcm11010092

**Published:** 2021-12-24

**Authors:** Kelsey Lipman, Anna Luan, Kimberly Stone, Irene Wapnir, Mardi Karin, Dung Nguyen

**Affiliations:** 1 Division of Plastic and Reconstruction Surgery, Stanford University, Stanford, CA 94304, USA; klipman@stanford.edu (K.L.); aluan@stanford.edu (A.L.); 2 Division of Breast Surgery, Stanford University, Stanford, CA 94304, USA; kstone@stanford.edu (K.S.); wapnir@stanford.edu (I.W.); mkarinmd@stanford.edu (M.K.)

**Keywords:** lymphedema prevention, LYMPHA, lymphaticovenous anastomosis

## Abstract

While surgical options exist to treat lymphedema after axillary lymph node dissection (ALND), the lymphatic microsurgical preventive healing approach (LYMPHA) has been introduced as a preventive measure performed during the primary surgery, thus avoiding the morbidity associated with lymphedema. Here, we highlight details of our operative technique and review postoperative outcomes. For our patients, limb measurements and body composition analyses were performed pre- and postoperatively. Intraoperatively, axillary reverse lymphatic mapping was performed with indocyanine green (ICG) and lymphazurin. SPY-PHI imaging was used to visualize the ICG uptake into axillary lymphatics. Cut lymphatics from excised nodes were preserved for lymphaticovenous anastomosis (LVA). At the completion of the microanastomosis, ICG was visualized draining from the lymphatic through the recipient vein. A retrospective review identified nineteen patients who underwent complete or partial mastectomy with ALND and subsequent LYMPHA over 19 months. The number of LVAs performed per patient ranged between 1–4 per axilla. The operating time ranged from 32–95 min. There were no surgical complications, and thus far one patient developed mild lymphedema with an average follow up of 10 months. At the clinic follow up, ICG and SPY angiography were used to confirm intact lymphatic conduits with an uptake of ICG across the axilla. This study supports LYMPHA as a feasible and effective method for lymphedema prevention.

## 1. Introduction

In the United States, lymphedema occurs most commonly as a sequelae of oncologic therapy [1,2]. It has been estimated that approximately 30–47% of patients who undergo mastectomy with axillary lymph node dissection (ALND) develop lymphedema postoperatively, with even higher rates reported in those who undergo adjuvant radiation therapy [3,4]. The development of lymphedema carries both physical and psychological burdens such as pain in the affected limb, decreased strength, reduced function, and decreased self-confidence [5,6].

Both non-operative and operative therapies are available for the management of lymphedema in order to reduce its severity, but these options do not return the limb to the premorbid state [2,4]. Non-operative techniques such as compression garments and massage are often time-intensive and require strict patient compliance. While microsurgical approaches for management are available, such as lymphovenous bypass and vascularized lymph node transfers, these require a separate operation for the patient after lymphedema has already occurred [2,4]. Lymphatic microsurgical preventive healing approach (LYMPHA), on the other hand, is focused on lymphedema prevention by performing prophylactic bypass of transected lymphatics to nearby venous outflow tracts at the time of the oncologic operation. By preventing lymphedema during the primary surgery, LYMPHA has the potential to avoid the lifelong morbidity that often results from lymphedema.

In this study, we discuss our preliminary experience with LYMPHA in terms of patient outcomes and also emphasize the details of incorporating this procedure into clinical practice. We highlight the coordination between breast and plastic surgery colleagues for dissection, intraoperative lymphatic mapping, and postoperative monitoring.

## 2. Materials and Methods

A retrospective review was performed for all patients who underwent axillary lymph node dissection with complete or partial mastectomy for breast cancer and subsequent LYMPHA procedure from September 2019 to April 2021. All patients had biopsy-proven positive lymph nodes prior to surgery, and thus were planned for axillary dissection preoperatively. Circumferential limb measurements and personalized body composition analysis using bioelectrical impedance software (InBody, Biospace Co., Seoul, Korea) were performed pre- and post-operatively. Limb measurements were performed in standard 4 cm intervals, with the most distal measurement at the wrist and compared to the opposite side. The standard truncated cone formula was used to calculate limb volume and % excess volume of the relevant limb. Bioelectrical impedance analysis was used to calculate the Lymphedema Index (L-Dex) [7].

Reverse axillary lymphatic mapping was performed using indocyanine green (ICG) and the SPY-PHI fluorescence imaging system (Stryker, MI, USA) to visualize uptake of ICG into lymphatic channels. Approximately 10 cm from the axilla, 0.2 mL of ICG was injected intradermally at several sites into the proximal upper inner arm using a 30-gauge needle and an 1 mL syringe. Additional 0.2 mL injections were injected into the webspaces of the hand to visualize baseline superficial lymphatic drainage in the arm.

**Figure 1:**
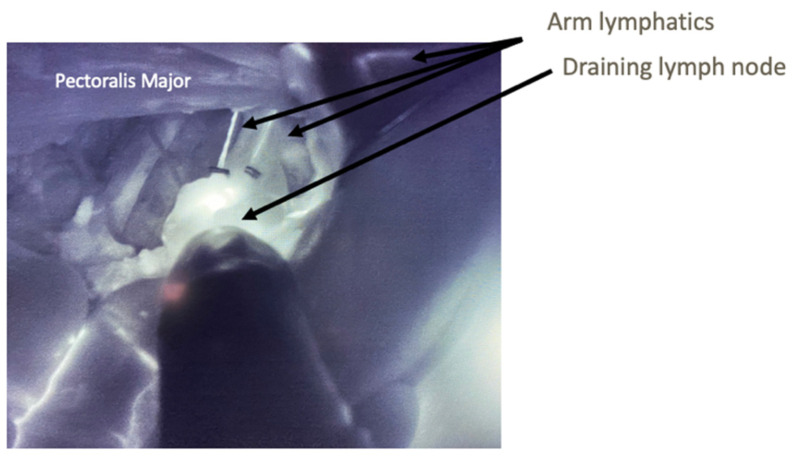
Intraoperative Lymphatic Mapping: Intraoperative view of left axillary lymph node dissection with ICG and SPY. Fluorescence allows for identification of arm lymphatics and draining lymph nodes. Clips are applied as shown to preserve as much length on the lymphatic as possible.

**Figure 2:**
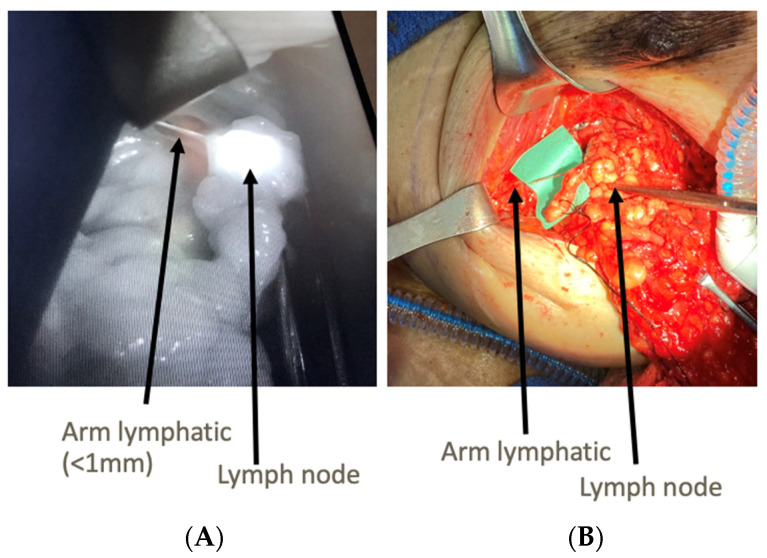
Identification of Structures during ALND: Right axillary lymph node dissection with pectoralis major retracted superiorly. (**A**) demonstrates a well-defined arm lymphatic draining into lymph node using SPY. (**B**) shows the “on the table” correlate during dissection.

**Figure 3:**
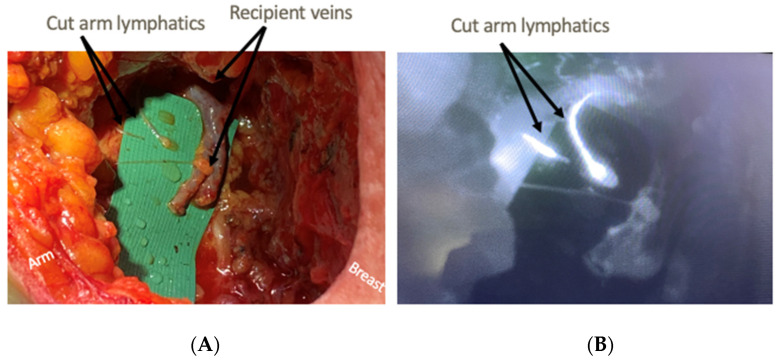
Preparation for LVA: (**A**) demonstrates two cut arm lymphatics and two nearby recipient veins, in this case branches of the pectoral vein. (**B**) demonstrates ICG and SPY correlate that shows bright lymphatics. The small structure in (**A**) that does not light up in (**B**) is a small nerve branch.

**Figure 4:**
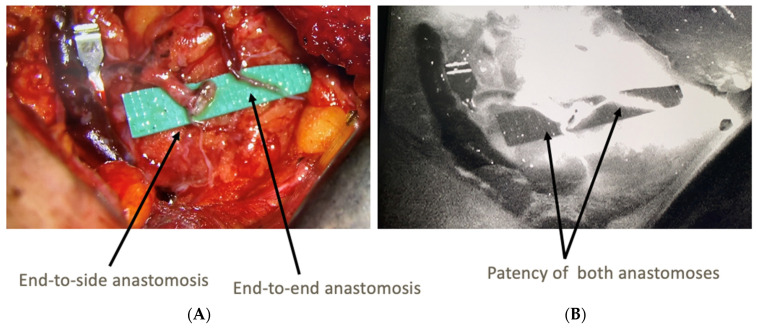
Evaluation of Anastomosis: (**A**) demonstrates two types of anastomoses, end-to-side and end-to-end, that can be performed depending on the size match between the lymphatic and recipient vein. After completion of the anastomoses, (**B**) shows patency of the LVAs using ICG and SPY.

The axillary dissections were performed in conjunction with breast surgery colleagues in order to assist in identification and preservation of lymphatics with sufficient length for lymphaticovenous anastomosis (LVA) (Figure 1). Figure 2 demonstrates how the use of ICG and SPY intraoperatively allows for identification of fluorescent lymphatics and nodes within the dissection bed. Without ICG and SPY, differentiation of structures, particularly small nerve branches versus lymphatics, is extremely difficult. Once the axillary dissection was complete and nodes were excised, lymphatics and recipient veins for LVA were identified (Figure 3). The lymphatics were typically < 1 mm in diameter. Based on relative size of the lymphatics and vein, anastomoses can be performed end-to-end or end-to-side (Figure 4A). The supermicroanastomoses were performed under the microscope at 10× magnification using interrupted 10-0 or 11-0 nylon sutures. At the completion of the microanastomosis, ICG was visualized draining through the veins (Figure 4B). The number of LVAs performed depends heavily on the location and available length of lymphatic channels and vein branches in the dissection bed.

Postoperative care and monitoring are critical to the success of the operation. Patients were instructed to wear custom fitted class I compression sleeves (and glove/gauntlet) for two weeks after surgery. They were instructed to remove the garment for showers and promptly replace. After two weeks, garment was worn as needed for high-risk activities such as nodal irradiation, flights, high altitude, and exercise. Patients were instructed to avoid raising the arm higher than 90 degrees (shoulder height) for 2 weeks postoperatively. After that time, they were encouraged to start gentle range of motion exercises. Those who did not reach full range of motion with independent stretching were referred to physical therapy. Patients were instructed to establish care with a lymphedema physical therapist immediately postoperatively to learn about lymphedema prevention exercises and techniques. L-Dex and circumferential limb measurements were performed at 3 months postoperatively.

Patient data including demographics, operative details, complications, limb measurements, and L-Dex values were recorded. The criteria for defining lymphedema remains inconsistent in the literature. In our population, lymphedema was defined as having both clinical signs/symptoms and an abnormal postoperative objective measure of lymphedema such as circumferential measurement or L-Dex. For objective measures, a volume difference between limbs of 10% or more and an L-Dex greater than 10 units from the baseline were considered abnormal. Postoperatively, ICG lymphangiography was performed in the clinic setting using the same injection technique described above to assess for the patency of the anastomoses.

## 3. Results

During our study period, 19 total patients successfully underwent LYMPHA at the time of ALND. The appropriate afferent lymphatics and recipient veins were identified in all cases, therefore there were no cases where the LYMPHA portion of the case was aborted. Table 1 summarizes the patient demographics for our patient population. The average age was 51.5 ± 14.1 years, and the average BMI was 26.7 ± 6.6 kg/m^2^. Six patients underwent unilateral mastectomy, five patients underwent bilateral mastectomy, and eight patients underwent unilateral partial mastectomy. A majority of the patients (16 of 19) underwent postoperative radiation therapy. Three patients underwent concomitant oncoplastic breast reduction, and eight patients underwent immediate breast reconstruction with either tissue expander placement (*n* = 5) or autologous reconstruction (*n* = 3). The average number of LVAs performed was 2.0 ± 0.9 (range 1–4). The operative time for the LVA portion of the case ranged from 32 to 95 min.

Patient outcomes are summarized in Table 2. The average follow up was 9.9 months. Patients were diagnosed with lymphedema if they had both clinical signs and symptoms consistent with lymphedema and at least one quantitative measurement consistent with lymphedema. One patient (Patient #13) developed mild clinical signs of lymphedema with an elevated L-Dex postoperatively. After noting early signs of lymphedema for this patient, several measures were immediately implemented including an increased daily use of pneumatic compression devices, increased manual lymphatic drainage with the lymphedema therapy team, and switching from a class I to class II compression garment to be worn at all times. Overall, there were no postoperative complications such as lymph leak, hematoma, seroma, or infection.

**Figure 5:**
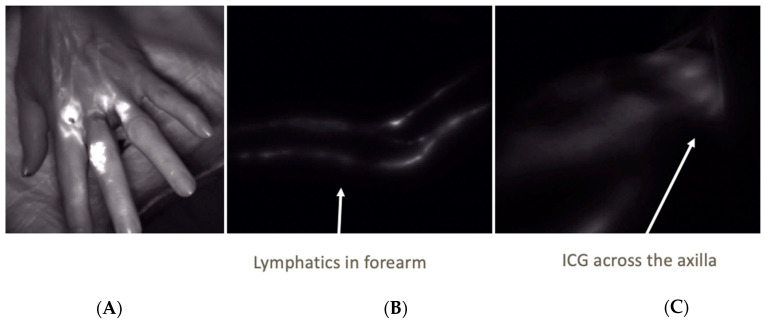
Lymphatic Mapping Postoperatively: ICG and SPY can be used postoperatively in clinic to assess patency of the anastomoses and evaluate lymphatic flow of the limb. (**A**) shows injection of ICG into the webspaces of the hand. The ICG is seen tracking proximally along the lymphatics across the forearm in (**B**). Eventually the ICG can be seen flowing across the axilla as in (**C**).

Postoperatively, ICG lymphangiography was used to confirm the patency of LVA in all patients. Figure 5 demonstrates the use of ICG and SPY-PHI in the clinic setting. Similar to the intraoperative lymphatic mapping, ICG is injected into the webspaces of the hand, tracked more proximally across the arm, and is eventually visualized crossing the axilla.

## 4. Discussion

Lymphedema remains a feared complication of breast cancer-related therapies that are critically important from an oncologic standpoint. Though current options exist for the management of lymphedema that can reduce severity, it is considered largely irreversible [4]. LYMPHA transitions the focus from lymphedema management toward prevention altogether.

The concept of axillary reverse lymphatic mapping emerged in the breast oncology literature in 2007 with the overall goal of preserving lymph nodes that drain the arm while removing those that drain the breast [8,9]. Nos et al. demonstrated the use of injecting blue dye in the arm to identify blue nodes and ducts in order to preserve arm lymphatic drainage [8]. However, this assumes that the upper extremity lymphatics are not involved in the metastasis of breast carcinoma and that the lymphatics of the breast and upper extremity are functionally and anatomically separate. A subsequent study by Pavlista et al. further examined the lymphatic anatomy of the axilla using blue dye in cadavers [10]. In this study, it was shown that the lymphatic drainage of the breast and upper extremity share connections in sentinel node groups in 24% of cases. As a result, a complete preservation of blue “arm” nodes in the axilla is not feasible from an oncologic standpoint [11]. However, the principles of axillary reverse mapping to identify lymphatics rather than nodes was subsequently applied by Boccardo et al. in 2009 to perform LYMPHA in the first series of patients [12].

In the initial study by Boccardo et al., LYMPHA was successfully performed in 18 of 19 patients using blue dye for lymphatic mapping and circumferential limb measurements for postoperative monitoring [12]. Using a diagnostic criterion of >1 cm discrepancy in arm measurements, no patients developed lymphedema. In a follow up study over the course of four years, Boccardo et al. reported a 4.05% rate of lymphedema among the 74 of 78 patients who successfully underwent LYMPHA during the study period [13].

Johnson et al. performed a meta-analysis to quantify the impact of radiation and LYMPHA on the incidence of lymphedema after ALND [4]. A total of 19 articles were used for the meta-analysis, three of which included patients who underwent LYMPHA [13,14,15]. LYMPHA was shown to reduce the risk of developing lymphedema after ALND in patients with or without adjuvant radiation. Subgroup analysis demonstrated an incidence of lymphedema of 14.1% in the ALND group vs. 2.1% in the ALND + LYMPHA group (*p* = 0.029). In the radiated subgroups, LYMPHA decreased the incidence of lymphedema from 33.4% to 10.3% (*p* = 0.004). Although the analysis supports the effectiveness of LYMPHA, it also highlights the marked heterogeneity in defining lymphedema and measuring it. Specifically, only 10.6% of the studies included used bioimpedance spectroscopy. In our study, we included both the traditional method of circumferential limb measurement, as well as bioimpedance spectroscopy, as it has been shown to be more sensitive for the detection of early lymphedema [16,17]. In addition to the combination of limb measurements and bioimpedance spectroscopy as previously described by Johnson et al., we also described the utility of postoperative ICG and SPY in clinic to assess the patency of lymphatic vessels, degree of dermal backflow, and lymphatic vessel contractility [18].

An additional source of variability in the literature is the method of lymphatic identification during axillary lymph node dissection. Previously described LYMPHA studies have used isosulfan blue dye, fluorescein isothiocyanate (FITC), and ICG [2,14,15]. As breast surgeons often utilize dual-tracer methods with technetium sulfur colloid as well as blue dye for sentinel node identification, alternative options for lymphatic identification are preferable. Both ICG and FITC offer alternatives to blue dye for lymphatic vessel mapping. At our institution, SPY is used for the visualization of ICG, while institutions that use FITC require a Pentero 900D microscope (Carl Zeiss Inc, Oberkochen, Germany) equipped with the YELLOW560 package for visualization [15,19]. Of important note, the depth of penetration of the ICG is approximately 4× that of FITC (20 mm versus 5 mm); therefore, it can be used for the transdermal visualization of lymphatic flow. As a result, ICG allows for intraoperative lymphatic mapping as well as the transdermal visualization of lymphatic flow at postoperative follow up in clinic.

As demonstrated in Table 2, patient #7 highlights the utility of performing two forms of objective monitoring postoperatively. This patient had mild clinical signs of lymphedema although the % volume difference on postoperative measurements remained within normal limits. However, an elevated L-Dex of 16.6 supports the notion that this patient may have early lymphedema based on the extracellular fluid measurements identified through bioimpedance spectroscopy. As a result, this particular patient’s measurements will be interesting to monitor over time. Patient #13, the patient who developed mild clinical signs of lymphedema with an elevated L-Dex postoperatively, highlights that even patients with successful LYMPHA procedures and confirmed intact lymphatic conduits on ICG remain at risk for lymphedema after surgery. Though the exact mechanism of her early lymphedema remains unclear, the normal ICG flow supports the likelihood that it is not secondary to a technical error in the anastomosis or postoperative fibrotic changes interfering with the anastomotic flow. Over time, it is possible that this patient may show abnormal ICG drainage patterns. In a study by Koelmeyer et al., compensatory drainage patterns of ICG in the upper and lower extremities have been used to guide personalized manual lymphatic drainage techniques [20]. If this patient were to develop abnormal ICG signaling, the results may help guide a personalized therapeutic intervention to maximize compensatory pathways.

The application of immediate lymphatic bypass has also recently expanded beyond the scope of breast cancer and has been applied to patients undergoing lymphadenectomy for melanoma and gynecologic malignancy [21,22,23]. Takeishi et al. performed intrapelvic LVA after lymph node dissection for patients with uterine cancer to prevent lower extremity lymphedema [23]. Cakmakoglu et al. performed immediate lymphatic reconstruction after ilioinguinal lymphadenectomy in patients with malignant melanoma [22]. As a result, as LYMPHA continues to be optimized in the breast cancer ALND population, the principles also have relevance for other subsets of oncologic surgery.

In our study, one patient simultaneously underwent immediate breast reconstruction with an omental free flap. As a result, for this patient, it may be difficult to determine the relative contributions of LYMPHA versus omental transfer on lymphedema prevention. In the intra-abdominal space, the omentum is known to serve a critical role in immune response and lymphatic drainage. Though our omental transfer for breast reconstruction did not involve the transfer of the gastric nodal basin or lymphovenous anastomosis of the efferent lymphatic that sometimes accompanies the gastroepiploic vessels, the omentum-associated lymph tissue (OALT) within the flap may have contributed partially to improved lymphatic drainage postoperatively [24]. For this patient, the flap reconstruction involved laparoscopic omental harvested by general surgery colleagues, augmenting the omentum with autologous fat, and placing the flap in an acellularized dermal matrix pocket for structural support. The omental tissue was not transferred specifically into the axilla. The gastroepiploic vessels were anastomosed to the internal mammary vessels. In the past, omental free flaps have demonstrated efficacy for the treatment of both upper and lower extremity lymphedema [25,26,27]. However, its use as a prophylactic measure has yet to be explored.

The highlights of this study include demonstrating the feasibility of LYMPHA, the use of ICG and SPY for intraoperative and postoperative lymphatic mapping, and the utility of bioimpedance spectroscopy for monitoring. The major limitations of our study include a nonrandomized design, a lack of a control group, and a limited follow up interval. A randomized, prospective study will be particularly useful to perform lymphatic mapping during ALND with and without LYMPHA to ensure that the variability in the axillary dissection technique does not alter lymphedema incidence itself. Regarding follow up duration, several studies have been performed in attempts to determine when the risk of lymphedema is highest after the treatment of breast cancer and which factors influence the time course. In a study by McDuff et al., for patients who received ALND without radiation, the hazard ratio of lymphedema development was greatest in the first 6 to 12 months after surgery [28]. For patients who underwent ALND with adjuvant radiation, the hazard ratio peaked between 18 to 24 months, thus linking radiation to a later onset of lymphedema likely due to the delayed effects of the radiation-induced fibrosis. As a result, for our patients who underwent ALND without radiation, the first postoperative year remains of the highest clinical importance. For the patients who underwent ALND with radiation, additional clinical follow up beyond this preliminary report of our outcomes will be useful. However, additional studies have shown that even small differences in early postoperative volume measurements may be predictive of long-term outcomes. Specht et al. performed a prospective study that demonstrated that low level volume changes within three months of surgery were significantly predictive of the development of lymphedema [29]. As a result, we advocate that even data within the first year postoperatively, particularly when able to assess bioimpedance spectroscopy, remains useful. Also, given that the study by McDuff et al. quantified lymphedema through arm volume measurements alone, it is possible that bioimpedance spectroscopy, which has been shown to be improved for the detection of early lymphedema, also makes the early clinical data useful for the radiated population. Regardless, additional studies with long-term follow ups can help to assess the longevity of LYMPHA in the future.

## 5. Conclusions

Our study further supports the current evidence that LYMPHA is safe, feasible, and effective for lymphedema prevention after axillary lymph node dissection. Though implementing the technique has an initial learning curve from both the breast and microsurgeon perspective, this paper aimed to provide a detailed description of our technique and the use of ICG lymphangiography and bioimpedance spectroscopy. As the technology used for the quantification of lymphedema becomes standardized and long-term data becomes available, further studies will determine if LYMPHA becomes a standard of care adjunct to ALND in the future.

## Figures and Tables

**Table 1:** Patient Demographics.

Total Number of Patients	19
Age (mean ± SD, years)	51.5 ± 14.1
BMI (mean ± SD, kg/m^2^)	26.7 ± 6.6
Oncologic Surgery	
Unilateral Mastectomy	6
Bilateral Mastectomy	5
Unilateral Partial Mastectomy	8
Adjuvant Radiation Therapy	16
Reconstructive Surgery	
TE/ADM	5
Abdominal Free Flap	2
Omental Free Flap	1
Oncoplastic breast reduction	3
Number of LVA (mean ± SD)	2.0 ± 0.9 (range 1–4)
Operative time for LVA (minutes)	32–95

TE, tissue expander; ADM, acellular dermal matrix.

**Table 2:** Postoperative Outcomes.

Patient	Number of LVA	Follow Up (Months)	Post-Op %Excess Volume	Post-Op L-Dex
1	1	21.3	15	9.5
2	2	20.4	3	3.5
3	2	17.8	−3	−3.8
4	2	15.3	0	0.1
5	4	14.0	−3	1.5
6	2	13.2	−3	2.8
7	1	9.6	8	16.6
8	3	9.0	−7	−2.1
9	3	7.7	−1	2.7
10	1	5.7	−1	−2.2
11	2	5.8	−9	1.8
12	3	7.9	3	−3.0
13	2	7.3	11	13.0
14	1	6.4	0	−0.5
15	1	5.4	−5	−5.3
16	2	5.5	2	2.6
17	3	5.3	5	−1.1
18	2	5.4	−9	1.0
19	1	4.4	−3	5.1

## Data Availability

The data for this article is not from a publicly archived dataset. The data presented in this study is available on request from the corresponding author.
